# Intestine‐Decipher Engineered Capsules Protect Against Sepsis‐induced Intestinal Injury via Broad‐spectrum Anti‐inflammation and Parthanatos Inhibition

**DOI:** 10.1002/advs.202412799

**Published:** 2025-01-21

**Authors:** Yan Yan, Bin Li, Qiuxia Gao, Miao Wu, Hua Ma, Jiawei Bai, Chengtai Ma, Xinyu Xie, Yuan Gong, Lingqi Xu, Xiaoxue Li, Wei Wang, Yanqiu Wu, Jiamei Wang, Huanhuan Wang, Yi Feng, Yunlong Zhang, Peiran Li, Huimin Shi, Fei Ma, Yue Jia, Han Duan, Xinting Fu, Wenying Wang, Liying Zhan, Xianjin Du, Huiting Zhou, Yuhui Liao

**Affiliations:** ^1^ Department of Critical Care Medicine Renmin Hospital of Wuhan University Wuhan 430060 China; ^2^ School of Inspection Ningxia Medical University Yinchuan Ningxia 750004 China; ^3^ Institute for Engineering Medicine Kunming Medical University Kunming 650500 China; ^4^ Institute of Pediatric Research Children's Hospital of Soochow University Suzhou 215025 China; ^5^ Molecular Diagnosis and Treatment Center for Infectious Diseases Dermatology Hospital of Southern Medical University Guangzhou Guangdong 510091 China; ^6^ Department of Microbiology School of Public Health Southern Medical University Guangzhou 510515 China

**Keywords:** broad‐spectrum anti‐inflammation, olaparib, parthanatos, pH‐responsive capsules, sepsis‐induced intestinal injury

## Abstract

Sepsis is a severe systemic inflammatory syndrome characterized by a dysregulated immune response to infection, often leading to high mortality rates. The intestine, owing to its distinct structure and physiological environment, plays a pivotal role in the pathophysiology of sepsis. It functions as the “central organ” or “engine” in the progression of sepsis, with intestinal injury exacerbating the condition. Despite the availability of current therapies that offer partial symptom relief, they fall short of adequately protecting the intestinal barrier. In this study, an advanced nanodrug formulation (OLA@MΦ NPs) is developed by coating macrophage membranes onto polymeric organic nanoparticles encapsulating olaparib. When loaded into pH‐responsive capsules, an intestine‐decipher engineered capsule (cp‐OLA@MΦ NPs) is successfully formulated. Upon oral administration in septic mice, these capsules withstand gastric acid and release their contents in the intestine, specifically targeting injured tissues. The released OLA@MΦ NPs effectively neutralize pro‐inflammatory cytokines via macrophage membrane receptors, while olaparib inhibits intestinal epithelial parthanatos (a form of programmed cell death) by suppressing poly(ADP‐ribose) polymerase 1 (PARP1) activation. This strategy significantly reduces bacterial translocation, slows the progression of sepsis, and enhances survival in septic mice, thus presenting a promising therapeutic approach for sepsis in clinical applications.

## Graphical abstract

This study developed a pH‐responsive drug, cp‐OLA@MΦ NPs, designed to target the intestine and release nanoparticles within the intestinal environment. By neutralizing pro‐inflammatory cytokines, inhibiting intestinal epithelial cell parthanatos, and maintaining intestinal barrier integrity, it significantly alleviates intestinal damage, reduces bacterial translocation, and markedly improves the survival rate of septic mice, offering new hope and a potential strategy for the treatment of sepsis.

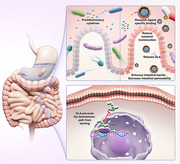

## 1 Introduction

Sepsis is a severe condition triggered by a dysregulated immune response to infection, leading to organ dysfunction and posing a substantial threat to human health.^[^ ^1^ ^]^ In 2017, global cases of sepsis reached ≈ 48.9 million, with 11 million related deaths, accounting for 19.7% of total global mortality.^[^ ^2^ ^]^ As a result, sepsis has become a critical public health challenge worldwide.

The intestine plays a pivotal role in sepsis pathophysiology due to its distinct physiological environment and structure.^[^ ^3^ ^]^ During sepsis, increased intestinal epithelial cell apoptosis, enhanced intestinal barrier permeability, disruption of mucosal integrity, and dysbiosis contribute to the onset of secondary intestinal‐origin infections.^[^ ^4^ ^]^ Intestinal barrier dysfunction, therefore, is considered a major driver of sepsis progression, with the intestine often referred to as the “central organ” or “engine” of sepsis.^[^ ^5^ ^]^ Maintaining and restoring intestinal barrier function is thus critical in sepsis management.^[^ ^6^ ^]^ However, conventional therapies, including fluid resuscitation, antimicrobial treatment, anticoagulants, and organ support, despite their overall benefits, fail to sufficiently protect the intestinal barrier.^[^ ^7^ ^]^ This underscores the urgent need for innovative strategies to effectively preserve the intestinal barrier in septic patients.

Parthanatos, a form of programmed cell death triggered by DNA damage, involves the overactivation of poly(ADP‐ribose) polymerase 1 (PARP‐1), resulting in the accumulation of poly(ADP‐ribose) (PAR) in the cytoplasm. This accumulation alters mitochondrial permeability, leading to the release of apoptosis‐inducing factor (AIF). AIF translocates to the nucleus, where it induces chromatin condensation and DNA fragmentation, ultimately culminating in cell death.^[^ ^8^ ^]^ Parthanatos has been implicated in various diseases, including neurodegenerative disorders (such as Parkinson's and Alzheimer's diseases),^[^ ^9^ ^]^ stroke,^[^ ^10^ ^]^ skin inflammation,^[^ ^11^ ^]^ diabetes,^[^ ^12^ ^]^ and pulmonary hypertension.^[^ ^13^ ^]^ However, its role in sepsis‐induced intestinal injury remains underexplored.

Olaparib (OLA), a PARP‐1 inhibitor, can prevent cell death via parthanatos.^[^ ^14^ ^]^ While its therapeutic potential in cancer has been extensively studied,^[^ ^15^ ^]^ its effects on sepsis‐induced intestinal injury are yet to be investigated. Exploring the impact of OLA on sepsis‐related intestinal damage and its potential to modulate parthanatos may provide new avenues for sepsis treatment. This study evaluated whether OLA can mitigate intestinal damage by inhibiting parthanatos, thereby preserving intestinal barrier function and improving disease outcomes in a sepsis model.

**Scheme 1:**
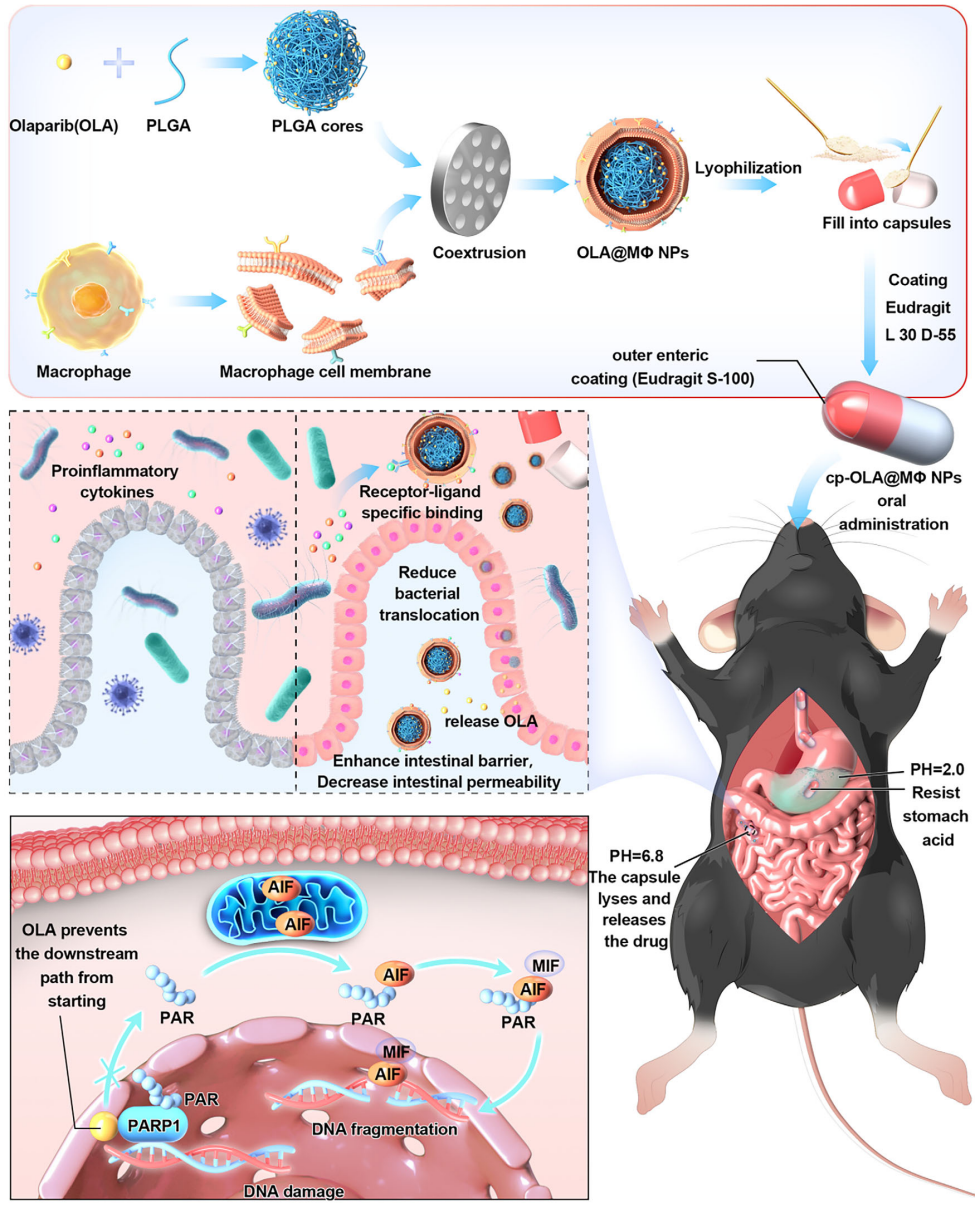
Schematic diagram of the preparation process of OLA@MΦ NPs and cp‐OLA@MΦ NPs and their therapeutic mechanism in sepsis‐induced intestinal injury mice.

A multistep nanoparticle (NP) drug, cp‐OLA@MΦ NP, was developed to test this hypothesis. Initially, OLA was encapsulated within poly(lactic‐co‐glycolic acid) (PLGA) NPs via the nanoprecipitation method to create OLA@NPs.^[^ ^16^ ^]^ Subsequently, the macrophage membrane was coated onto the PLGA core using a coextrusion method to generate OLA@MΦ NPs.^[^ ^17^ ^]^ The macrophage membrane is rich in receptors for inflammatory factors, including interleukin – 1 receptor (IL‐1R), interleukin – 6 receptor (IL‐6R), and tumor necrosis factor receptor (TNFR), as well as Toll‐like receptors (TLRs) such as TLR4. These receptors enable broad neutralization of proinflammatory cytokines, thereby attenuating the inflammatory response.^[^ ^18^ ^]^ After lyophilization, OLA@MΦ NPs were encapsulated in gelatin capsules and coated with the pH‐responsive material EUDRAGIT L 30 D‐55, resulting in the final cp‐OLA@MΦ NPs formulation (**Scheme** **1**). The EUDRAGIT L 30 D‐55 coating effectively shields OLA@MΦ NPs from degradation in the acidic, enzymatic environment of the stomach, ensuring targeted release in the intestine.^[^ ^19^ ^]^

Experimental results demonstrated that cp‐OLA@MΦ NPs effectively neutralized proinflammatory cytokines, inhibited intestinal epithelial apoptosis, preserved intestinal barrier integrity, reduced bacterial translocation, and significantly alleviated sepsis symptoms (Scheme 1). In summary, cp‐OLA@MΦ NPs hold significant promise as both a therapeutic agent for sepsis and a novel therapeutic strategy for future clinical applications.

## 2 Results

### 2.1 Preparation and Characterization of cp‐OLA@MΦ NPs

**Figure 1:**
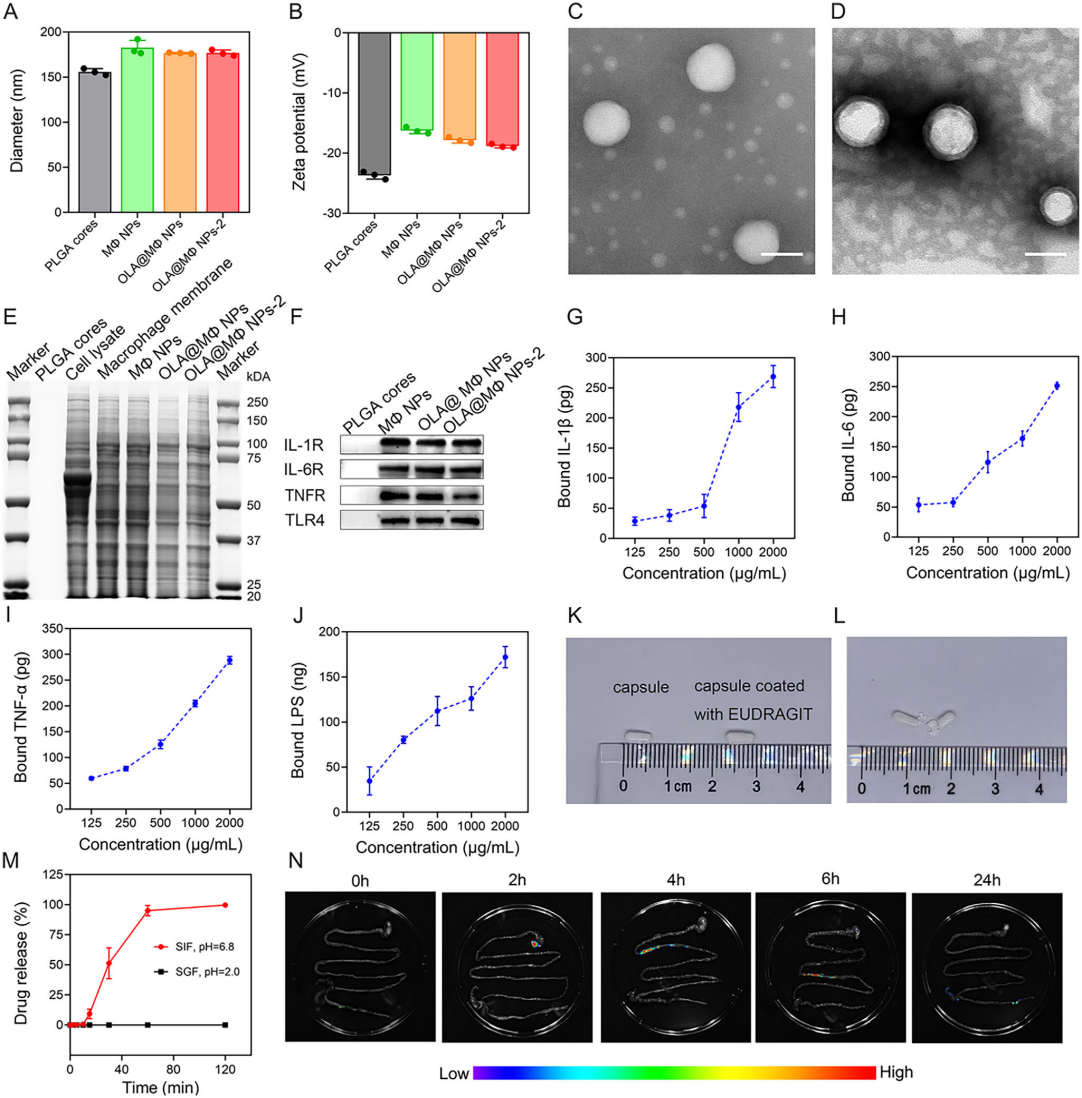
Characterization of OLA@MΦ NPs and cp‐OLA@MΦ NPs. A) Hydrodynamic sizes of PLGA cores, MΦ NPs, OLA@MΦ NPs, and OLA@MΦ NPs‐2 (OLA@MΦ NPs isolated from cp‐OLA@MΦ NPs) (*n* = 3). B) Zeta potential of PLGA cores, MΦ NPs, OLA@MΦ NPs, and OLA@MΦ NPs‐2 (*n* = 3). C,D) TEM images of PLGA cores (C) and OLA@MΦ NPs‐2 (D). Scale bar = 50 nm. E) SDS–PAGE analysis of protein components in PLGA cores, cell lysate, macrophage membranes, MΦ NPs, OLA@MΦ NPs, and OLA@MΦ NPs‐2. F) Western blot analysis of IL‐1R, IL‐6R, TNFR, and TLR4 in PLGA cores, MΦ NPs, OLA@MΦ NPs, and OLA@MΦ NPs‐2. Samples were evaluated at the same protein concentration. G‐I) Binding of OLA@MΦ NPs‐2 to inflammatory cytokines IL‐1β (500 pg), IL‐6 (500 pg), and TNF‐α (500 pg) at different concentrations (*n* = 4). J) Binding of OLA@MΦ NPs‐2 to LPS (300 ng) at varying concentrations (*n* = 4). K,L) Images of a capsule loaded with OLA@MΦ NPs and a capsule coated with EUDRAGIT (cp‐OLA@MΦ NPs) (K), and an opened capsule (L). M) Release of OLA@MΦ NPs from capsules coated with EUDRAGIT L 30‐D55 in simulated gastric fluid (SGF, pH = 2, black) and simulated intestinal fluid (SIF, pH = 6.8, red) (*n* = 3). N) Gastrointestinal imaging of mice after the oral administration of DiR‐labeled cp‐OLA@MΦ NPs at different time points. Data are presented as mean ± SD.

A series of tests were performed to assess the structural integrity and characterization of the prepared nanoparticles. Dynamic light scattering measurements showed that the freshly prepared MΦ NPs, OLA@MΦ NPs, and resuspended cp‐OLA@MΦ NPs exhibited similar hydrodynamic diameters (182.48, 176.63, and 176.70 nm, respectively) (**Figure** **1**A) and surface zeta potential values (−16.23, −17.83, and −18.81 mV, respectively) (Figure 1B), indicating minimal impact of the lyophilization and encapsulation processes on nanoparticle structure. Additionally, the particle size increased from 155.75 nm for the PLGA core to 176.70 nm for cp‐OLA@MΦ NPs, confirming the successful integration of the cell membrane onto the PLGA core (Figure 1A). Transmission electron microscopy (TEM) further corroborated this finding, with cp‐OLA@MΦ NPs exhibiting a distinctive core‐shell structure compared to the bare PLGA core (Figure 1C,D).

Following membrane coating, cp‐OLA@MΦ NPs retained key protein receptors and biological characteristics of macrophages. Sodium dodecyl sulfate–polyacrylamide gel electrophoresis (SDS‐PAGE) analysis revealed that the protein profile of cp‐OLA@MΦ NPs was comparable to that of pure macrophage membranes, MΦ NPs, and OLA@MΦ NPs, but distinct from macrophage lysate, which contains intracellular proteins. This confirms that only cell membrane components were encapsulated in cp‐OLA@MΦ NPs (Figure 1E). Previous studies have shown that macrophages express various proinflammatory cytokine receptors (such as IL‐1β, IL‐6, and TNF‐α receptors) and TLRs (such as TLR4) on their surface, enabling the adsorption of proinflammatory cytokines and Lipopolysaccharide (LPS).^[^ ^20^ ^]^ Western blot analysis demonstrated that cp‐OLA@MΦ NPs retained TLR4 for LPS adsorption, as well as IL‐1R, IL‐6R, and TNFR for proinflammatory cytokine binding (Figure 1F). Furthermore, coincubation of OLA@MΦ NPs released from the capsules with IL‐1β, IL‐6, TNF‐α, and LPS, key mediators of the cytokine storm during sepsis, showed dose‐dependent binding of the NPs to these cytokines (Figure 1G–J).^[^ ^21^ ^]^

The drug‐loaded gelatin capsule, ≈ 0.5 cm in length, showed no significant change in size after being coated with EUDRAGIT L 30‐D55 (Figure 1K,L). To evaluate the pH‐responsive behavior of the capsule, cp‐OLA@MΦ NPs were labeled with DiR and incubated in simulated gastric fluid (SGF, pH = 2.0). The results showed that minimal OLA@MΦ NPs were released within the first 120 min. However, when placed in simulated intestinal fluid (SIF, pH = 6.8), over 90% of the OLA@MΦ NPs were released within 60 min, and complete release occurred after 120 min (Figure 1M). This confirmed the pH‐responsive release of OLA@MΦ NPs from the capsule.

To investigate the intestinal targeting of cp‐OLA@MΦ NPs, fluorescent‐labeled cp‐OLA@MΦ NPs were orally administered to C57BL/6 mice, and their gastrointestinal distribution was tracked at predetermined time points (Figure 1N). Two hours post‐administration, cp‐OLA@MΦ NPs were primarily localized in the stomach, with the fluorescence signal confined to a narrow region, indicating the intact structure of the capsule. After 4 h, a broader fluorescence distribution was observed in the intestines, suggesting that the capsule had dissolved, allowing the release of cp‐OLA@MΦ NPs into the intestinal tract.

### 2.2 OLA@MΦ NPs Inhibit Parthanatos in Intestinal Epithelial Cells

The protective effect of OLA@MΦ NPs on IEC‐6 cells through the inhibition of parthanatos was investigated. In sepsis, excessive production of pro‐inflammatory cytokines and reactive oxygen species (ROS),^[^ ^22^ ^]^ including hydrogen peroxide (H_2_O_2_), singlet oxygen (^1^O_2_), and superoxide anion (·O^2−^),^[^ ^23^ ^]^ contributes to mitochondrial dysfunction, cell death, and multiple organ failure.^[^ ^24^ ^]^ Overproduction of ROS is central to the pathogenesis of sepsis and the development of organ failure.^[^ ^25^ ^]^ H_2_O_2_, in particular, is secreted by several bacterial pathogens, such as *Streptococcus pneumoniae*, *Streptococcus oralis*, and *Pseudomonas aeruginosa*,^[^ ^26^ ^]^ which are implicated in sepsis or can induce it under certain conditions. Parthanatos has been shown to be triggered by ischemia, ROS, alkylating agents, and ultraviolet radiation.^[^ ^27^ ^]^ H_2_O_2_ was employed to induce parthanatos in intestinal epithelial cells, mimicking this pathogenic mechanism.

**Figure 2:**
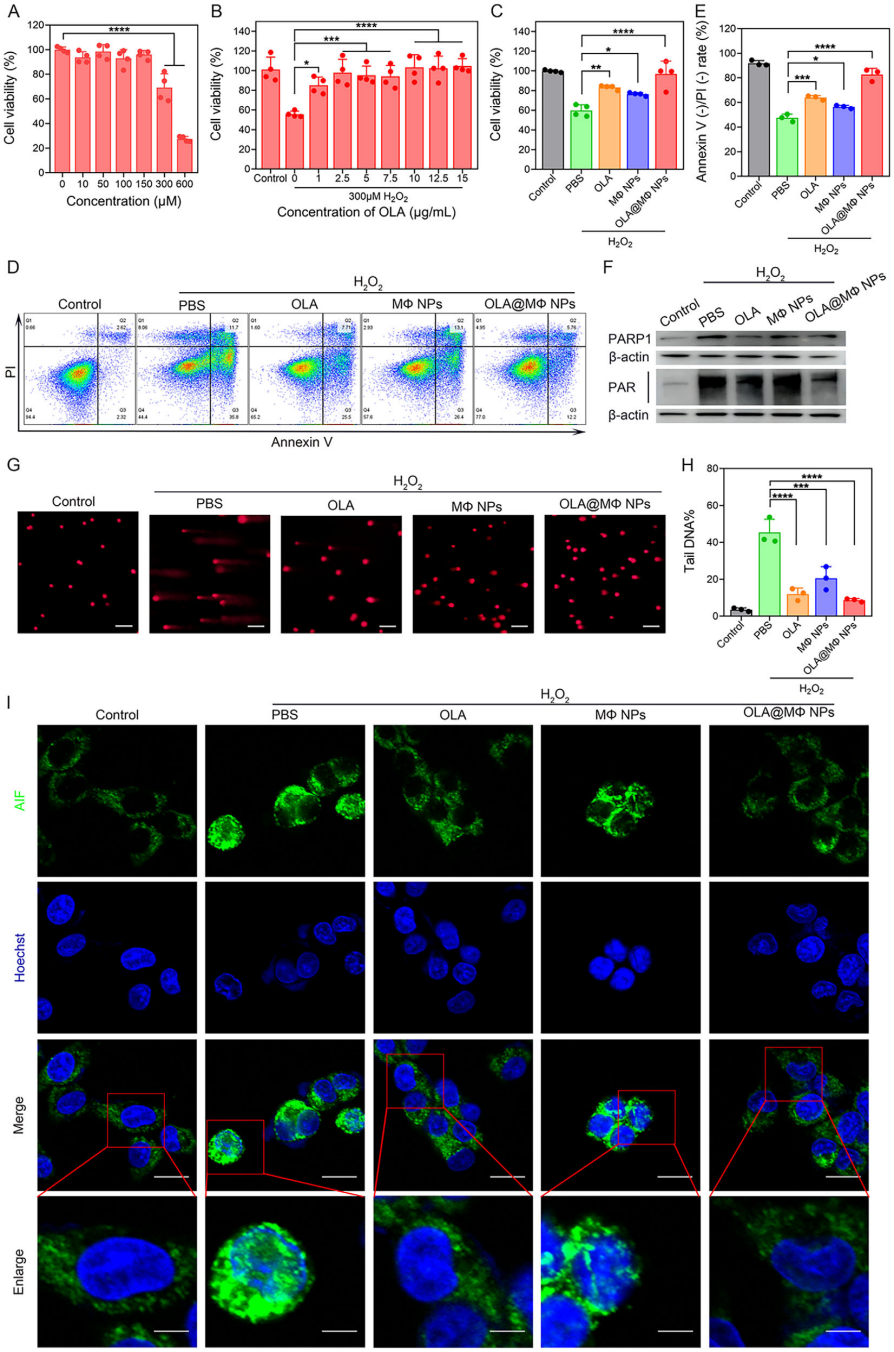
OLA@MΦ NPs inhibit parthanatos in intestinal epithelial cells. A) Determination of the viability of IEC‐6 cells following treatment with different concentrations of H_2_O_2_ (0–600 µm) using CCK‐8 assay (*n* = 4). B) Effect of OLA (0–15 µg·mL⁻¹) on IEC‐6 cell viability after exposure to 300 µm H_2_O_2_ (*n* = 4). C) Viability of IEC‐6 cells after different treatments (*n* = 4). D) Flow cytometry analysis of Annexin V/PI staining in IEC‐6 cells. E) Quantification of cells stained with Annexin V (−)/PI (−) as shown in (D) (*n* = 3). F) Western blot analysis of PARP1 and PAR expression following 1 h of treatment under different conditions. G,H) Comet assay results and statistical analysis of IEC‐6 cells under different treatments (*n* = 3). Scale bar = 100 µm. I) AIF localization and nuclear translocation following different treatments. Scale bar = 20 µm (original image), Scale bar = 7 µm (enlarged view). Control group: none; PBS group: PBS + 300 µm H_2_O_2_; OLA group: 5 µg·mL⁻¹ OLA + 300 µm H_2_O_2_; MΦ NPs group: MΦ NPs + 300 µm H_2_O_2_; OLA@MΦ NPs group: OLA@MΦ NPs + 300 µm H_2_O_2,_ equivalent to 5 µg·mL⁻¹ OLA. Data are presented as mean ± SD. Statistical significance was calculated via ordinary one‐way ANOVA. *p < 0.05; **p < 0.01; ***p < 0.001; ****p < 0.0001.

IEC‐6 cells were exposed to varying concentrations of H_2_O_2_ (0–600 µm) to determine the lethal concentration for these cells. The data revealed a significant decrease in cell viability when H_2_O_2_ concentrations exceeded 300 µm (**Figure** **2**A). The protective effect of OLA (0–15 µg·mL⁻¹) against 300 µm H_2_O_2_‐induced damage in IEC‐6 cells was subsequently assessed (Figure 2B), showing that OLA notably alleviated the H_2_O_2_‐induced cell injury. Furthermore, OLA@MΦ NPs, equivalent to 5 µg·mL⁻¹ OLA, also effectively mitigated H_2_O_2_‐induced damage (Figure 2C). Flow cytometry analyses further confirmed that OLA@MΦ NPs significantly reduced the H_2_O_2_‐induced cell death in IEC‐6 cells (Figure 2D,E).

Cell damage activates PARP1, leading to PAR accumulation.^[^ ^28^ ^]^ OLA can reduce PAR production by inhibiting PARP1 activation. A significant reduction in PAR expression was observed after OLA treatment in IEC‐6 cells exposed to H_2_O_2_ for 1 h (Figure S1, Supporting Information). Western blot analysis confirmed that both OLA and OLA@MΦ NPs significantly downregulated PARP1 and PAR levels compared to the PBS group (Figure 2F). In vivo, elevated PARP1 and PAR expression was observed in the intestines of septic mice, while treatment with OLA, OLA@MΦ NPs, or cp‐OLA@MΦ NPs resulted in reduced PARP1 and PAR levels (Figure S2, Supporting Information). During parthanatos, AIF translocates to the nucleus, leading to chromatin condensation and DNA fragmentation, ultimately causing cell death.^[^ ^29^ ^]^ Comet assay results demonstrated that H_2_O_2_ induced DNA damage in IEC‐6 cells, with treatment using OLA, MΦ NPs, or OLA@MΦ NPs partially alleviating this damage (Figure 2G,H). Additionally, OLA and OLA@MΦ NPs effectively inhibited AIF nuclear translocation (Figure 2I).

### 2.3 OLA@MΦ NPs Alleviate Intestinal Injury and Improve Barrier Permeability in IEC‐6 Cells

**Figure 3:**
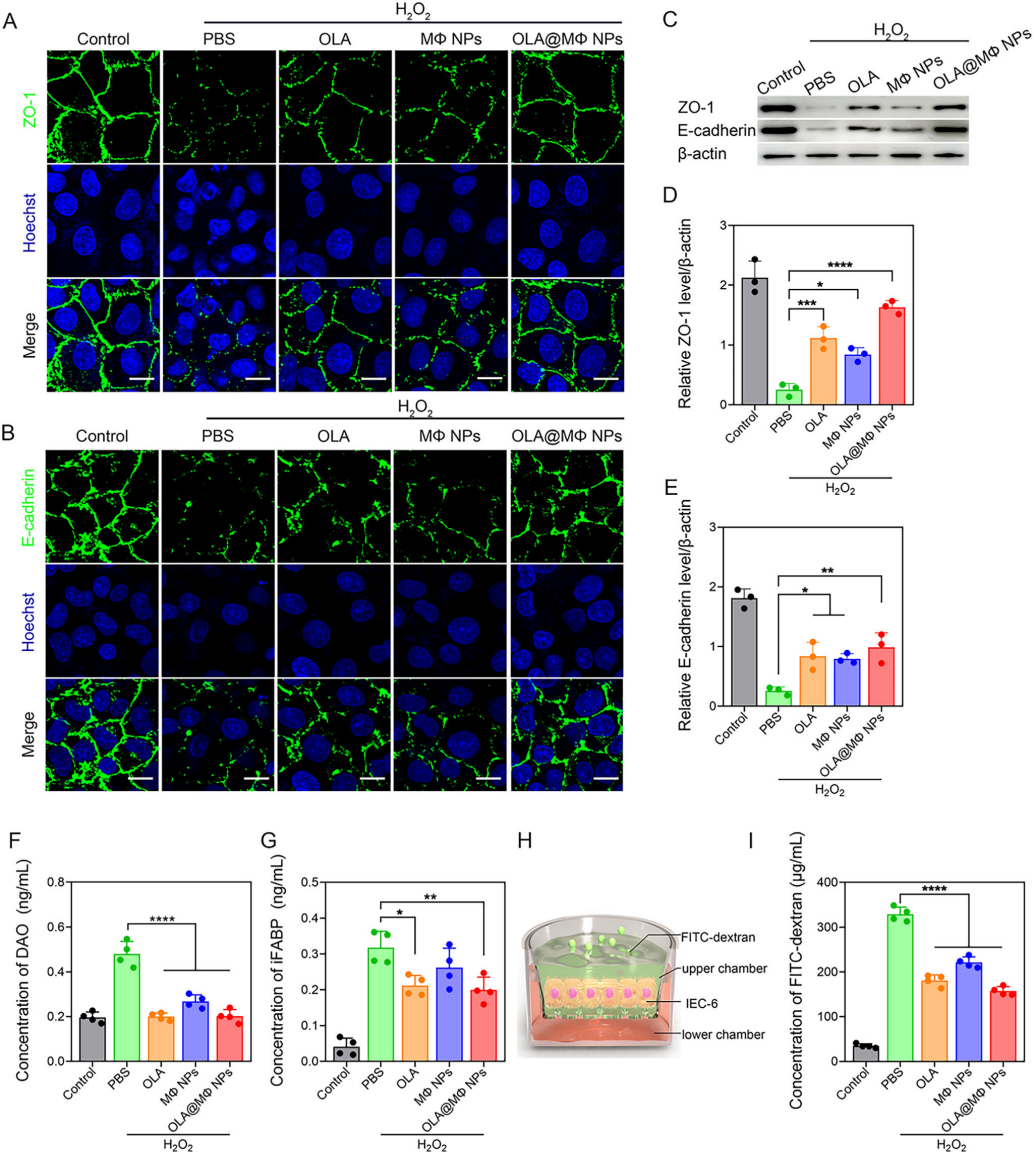
OLA@MΦ NPs alleviate intestinal injury and improve barrier permeability in IEC‐6 cells. A,B) Immunofluorescence staining for ZO‐1 and E‐cadherin in IEC‐6 cells treated with various conditions. Scale bar = 20 µm. C) Western blot analysis of ZO‐1 and E‐cadherin expression in IEC‐6 cells under different treatments. D,E) Quantification of ZO‐1 and E‐cadherin expression levels from (C) (*n* = 3). F,G) DAO and iFABP levels in the supernatant of IEC‐6 cells after different treatments (*n* = 4). H) Schematic representation of the Transwell experiment showing FITC‐dextran passing through the intestinal epithelial layer into the lower chamber. I) Quantification of FITC‐dextran in the lower chamber of the Transwell (*n* = 4). Control group: none; PBS group: PBS + 300 µm H_2_O_2_; OLA group: 5 µg·mL⁻¹ OLA + 300 µm H_2_O_2_; MΦ NPs group: MΦ NPs + 300 µm H_2_O_2_; OLA@MΦ NPs group: OLA@MΦ NPs + 300 µm H_2_O_2,_ equivalent to 5 µg·mL⁻¹ OLA. Data are presented as mean ± SD. Statistical significance was assessed using one‐way ANOVA. *p < 0.05; **p < 0.01; ***p < 0.001; ****p < 0.0001.

The effects of OLA@MΦ NPs on intestinal injury and barrier permeability in IEC‐6 cells were evaluated. Intestinal permeability is primarily regulated by tight junction proteins, such as ZO‐1 and E‐cadherin.^[^ ^30^ ^]^ Immunofluorescence analysis (**Figure** **3**A,B) showed intact and continuous ZO‐1 and E‐cadherin junctions in the control group. However, H_2_O_2_ stimulation resulted in a marked reduction in the expression of these proteins, with discontinuous staining patterns. In contrast, treatment with OLA, MΦ NPs, or OLA@MΦ NPs restored ZO‐1 and E‐cadherin expression, with a more linear distribution observed, especially in the OLA@MΦ NPs group, indicating superior efficacy. These results were further confirmed by Western blot analysis, which demonstrated a significant decrease in ZO‐1 and E‐cadherin expression in the PBS group, whereas their expression was significantly elevated following treatment with OLA, MΦ NPs, or OLA@MΦ NPs, with the highest increase observed in the OLA@MΦ NPs group (Figure 3C–E).

Furthermore, the levels of diamine oxidase (DAO) and intestinal fatty‐acid binding protein (iFABP), biomarkers for intestinal epithelial cell injury,^[^ ^31^ ^]^ were significantly elevated in the PBS group following H_2_O_2_ exposure but significantly decreased after treatment with OLA, MΦ NPs, or OLA@MΦ NPs (Figure 3F,G). The permeability of IEC‐6 cells was then assessed using a Transwell (Figure 3H), and the results (Figure 3I) showed a notable reduction in FITC‐dextran concentration in the lower chamber following treatment with OLA, MΦ NPs, or OLA@MΦ NPs, with reductions of 44.95%, 32.64%, and 52.13%, respectively.

### 2.4 Therapeutic Effect of Oral Administration of cp‐OLA@MΦ NPs in Septic Mice

**Figure 4:**
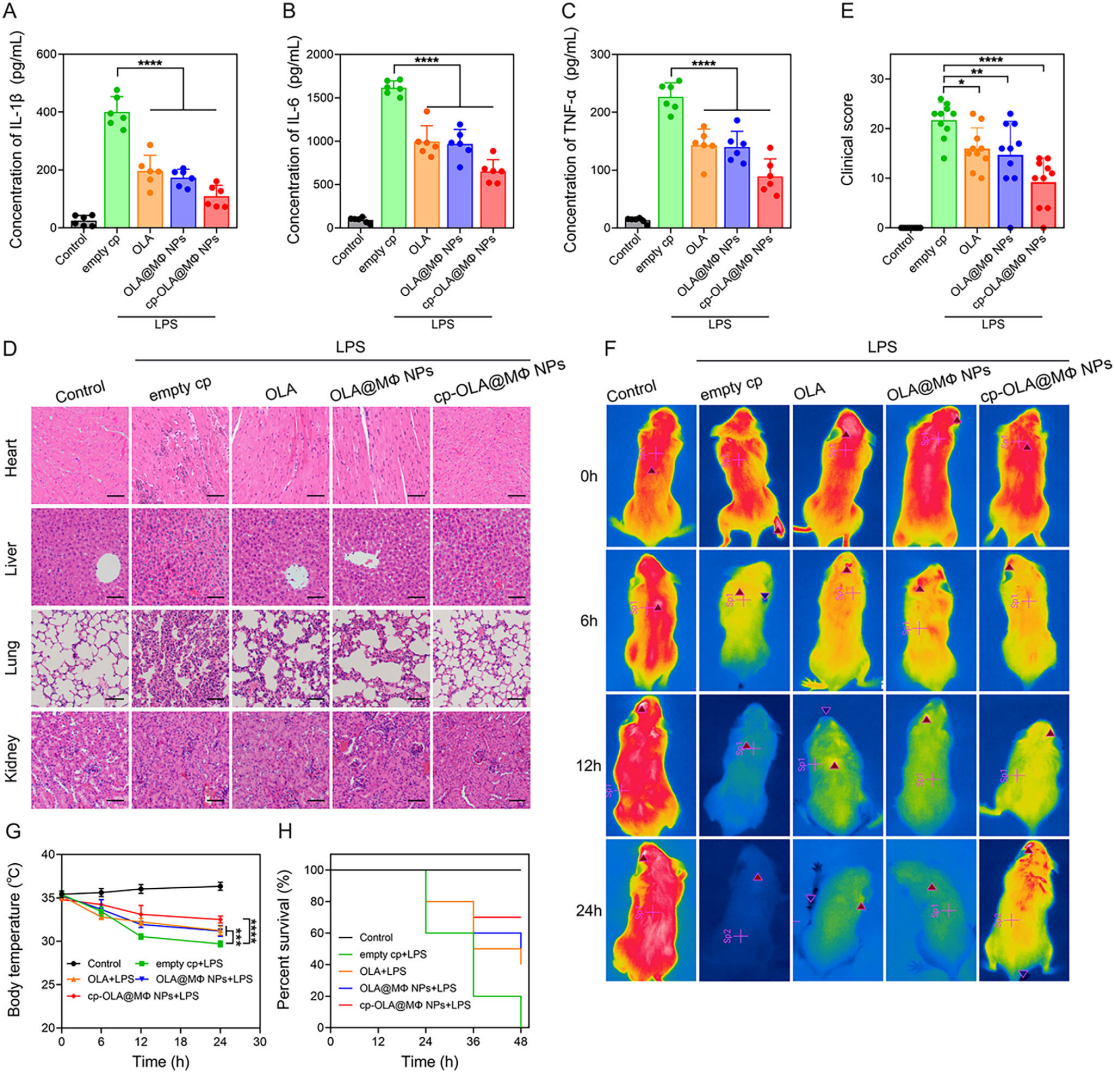
Therapeutic effect of oral administration of cp‐OLA@MΦ NPs in septic mice. A–C) Serum levels of proinflammatory cytokines IL‐1β, IL‐6, and TNF‐α 24 h after LPS injection (*n* = 6). D) HE staining of tissues from mice in different groups. Scale bar = 50 µm. E) Clinical scores of septic mice 24 h after treatment (*n* = 10). F,G) Infrared thermal imaging and body temperature detection of septic mice following various treatments (*n* = 5). H) Survival rates of septic mice following various treatments (*n* = 10). Data are presented as mean ± SD. Statistical significance was calculated using one‐way ANOVA. *p < 0.05; **p < 0.01; ***p < 0.001; ****p < 0.0001.

The therapeutic effects of cp‐OLA@MΦ NPs were evaluated in septic mice, which were randomly divided into five groups. The control group received an intraperitoneal injection of PBS, while the remaining groups received 15 mg·kg⁻¹ LPS to induce sepsis, followed by oral administration of an empty capsule (empty cp), OLA, OLA@MΦ NPs, or cp‐OLA@MΦ NPs (equivalent to 10 mg·kg⁻¹ OLA). Sepsis induces a large release of proinflammatory cytokines,^[^ ^32^ ^]^ and serum samples were collected for analysis 24 h after the model was established (**Figure** **4**A–C). The results showed that LPS injection significantly increased the levels of proinflammatory cytokines IL‐1β, IL‐6, and TNF‐α, confirming successful sepsis induction. After treatment with OLA, OLA@MΦ NPs, or cp‐OLA@MΦ NPs, the levels of these cytokines significantly decreased. Notably, the cytokine levels were lower in mice treated with cp‐OLA@MΦ NPs compared to those treated with OLA and OLA@MΦ NPs.

Sepsis often causes multiple organ failure. To assess the extent of organ damage, hematoxylin‐eosin (HE) staining was performed (Figure 4D). In the empty cp group, significant inflammatory cell infiltration was observed in various organs compared to the control group. Treatment with OLA, OLA@MΦ NPs, or cp‐OLA@MΦ NPs led to partial recovery of organ function, with reduced inflammation. The most pronounced tissue recovery was observed in the lungs of mice treated with cp‐OLA@MΦ NPs, where tissue damage was markedly improved.

Next, clinical scores and body temperature were evaluated as indicators of disease progression and immune‐mediated damage.^[^ ^33^ ^]^ Treatment with OLA, OLA@MΦ NPs, or cp‐OLA@MΦ NPs significantly reduced clinical scores, indicating an improvement in the septic condition of the mice (Figure 4E). LPS injection caused a dramatic drop in body temperature, which was alleviated by OLA, OLA@MΦ NPs, or cp‐OLA@MΦ NPs (Figure 4F,G). Remarkably, cp‐OLA@MΦ NPs showed the most effective protection, preventing LPS‐induced hypothermia. Additionally, cp‐OLA@MΦ NPs greatly improved survival rates. In the empty cp group, survival dropped to 0% after 48 h, whereas survival rates increased to 40%, 50%, and 70% in the OLA, OLA@MΦ NPs, and cp‐OLA@MΦ NPs groups, respectively (Figure 4H).

### 2.5 Impact of Oral Administration of cp‐OLA@MΦ NPs on Intestinal Injury in Septic Mice

**Figure 5:**
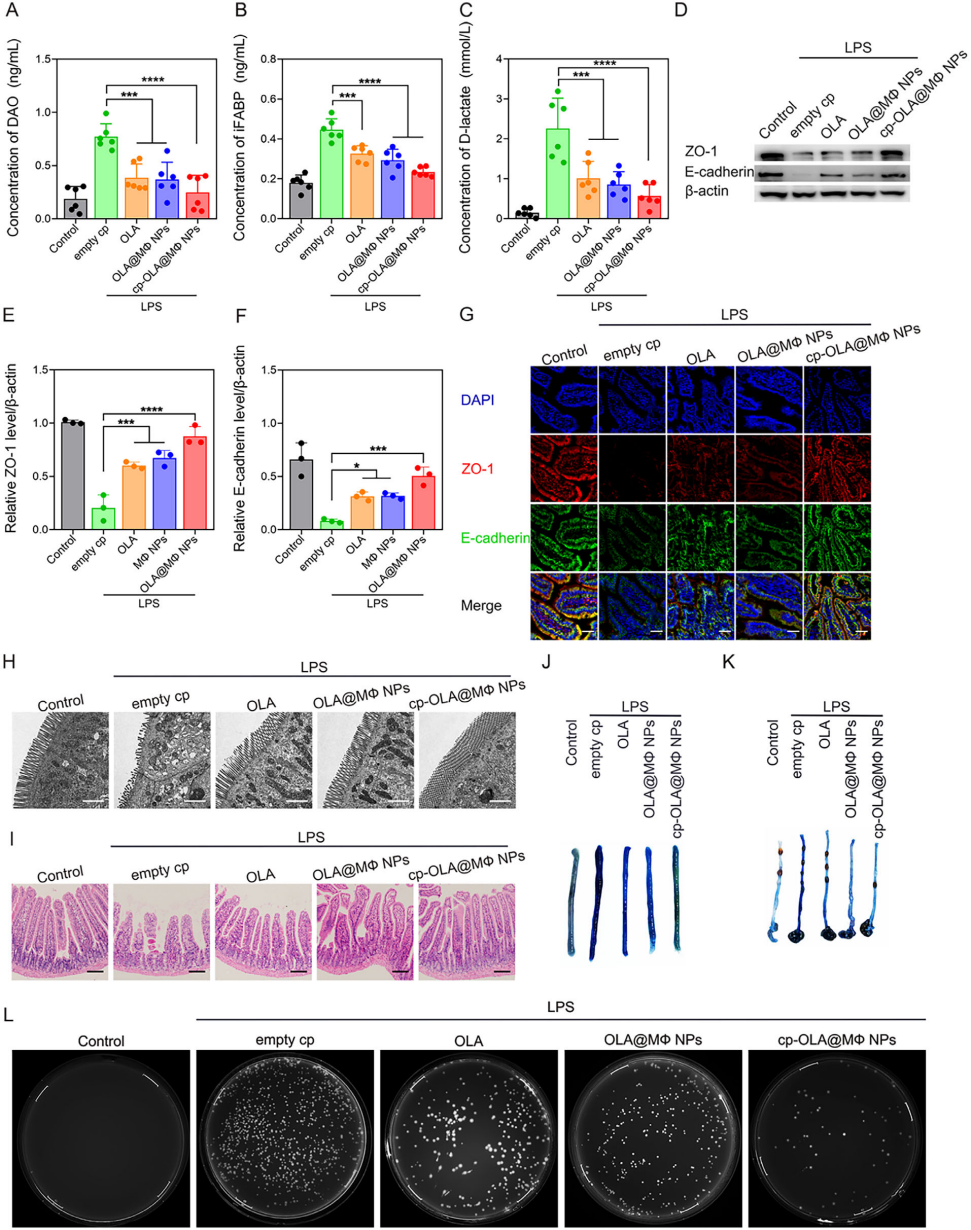
Impact of oral administration of cp‐OLA@MΦ NPs on intestinal injury in septic mice. A–C) Serum levels of DAO, iFABP, and D‐lactate after different treatments (*n* = 6). D) Western blot analysis of ZO‐1 and E‐cadherin in intestinal tissue. E,F) Quantification of ZO‐1 and E‐cadherin from (D) (*n* = 3). G) Immunofluorescence detection of ZO‐1 and E‐cadherin in mouse intestinal tissue. Scale bar = 50 µm. H) TEM images of the small intestine. Scale bar = 1.5 µm. I) Representative HE staining of mouse intestinal tissue. Scale bar = 100 µm. J,K) Evans blue leakage in the small intestine (J) and colon (K). L) Bacterial colonies in peritoneal lavage. Data are presented as mean ± SD. Statistical analysis was performed using one‐way ANOVA. *p < 0.05; **p < 0.01; ***p < 0.001; ****p < 0.0001.

The effects of cp‐OLA@MΦ NPs on intestinal barrier function and permeability were evaluated to determine their role in maintaining intestinal homeostasis in septic mice. In addition to DAO and iFABP, D‐lactate, a byproduct of bacterial metabolism and glycolysis in the intestine, serves as a critical marker of intestinal barrier integrity.^[^ ^34^ ^]^ As shown in **Figure** **5**A–C, sepsis significantly disrupts intestinal function. Serum levels of DAO, iFABP, and D‐lactate were markedly elevated in the empty cp group compared to the control group. However, these markers were notably reduced after treatment, particularly in the cp‐OLA@MΦ NPs group, highlighting the enhanced ability of cp‐OLA@MΦ NPs to alleviate intestinal damage induced by sepsis.

The impact on tight junctions in the intestinal epithelium was next assessed. Western blot analysis of intestinal tissues revealed a significant reduction in ZO‐1 and E‐cadherin expression in the empty cp group. Treatment with OLA, OLA@MΦ NPs, or cp‐OLA@MΦ NPs led to a substantial increase in these tight junction proteins, with the most pronounced upregulation observed in the cp‐OLA@MΦ NPs group (Figure 5D–F). This trend was further corroborated by immunofluorescence results (Figure 5G). TEM analysis (Figure 5H and Figure S3, Supporting Information) revealed well‐organized microvilli with narrow intercellular spaces and intact morphology in the control group. In contrast, the empty cp group exhibited sparse, disorganized microvilli with widened intercellular spaces and significant ultrastructural damage, including swollen and ruptured mitochondria. Treatment with OLA, OLA@MΦ NPs, or cp‐OLA@MΦ NPs ameliorated these damages to varying degrees, with the cp‐OLA@MΦ NPs group showing the most significant improvement.

HE staining (Figure 5I) revealed a marked reduction in villus length, increased thickness, and disorganization in the sepsis model. The epithelial surface exhibited significant villus loss, with extensive detachment of the intestinal mucosal epithelium from the lamina propria and notably expanded subepithelial spaces. In contrast to the empty cp group, treatment groups displayed substantial improvements in villus architecture, particularly in the cp‐OLA@MΦ NPs group.

The effect of sepsis on intestinal barrier integrity was further assessed by measuring Evans blue leakage and bacterial load in the peritoneal cavity. Compared to the empty cp group, OLA, OLA@MΦ NPs, and cp‐OLA@MΦ NPs treatments reduced Evans blue leakage, with the cp‐OLA@MΦ NPs group demonstrating the most pronounced effect (Figure 5J,K). Moreover, the bacterial burden in the peritoneal cavity was significantly lower in treated groups than in the empty cp group, particularly in the cp‐OLA@MΦ NPs group (Figure 5L).

### 2.6 Biosafety Assessment of OLA@MΦ NPs and cp‐OLA@MΦ NPs

**Figure 6:**
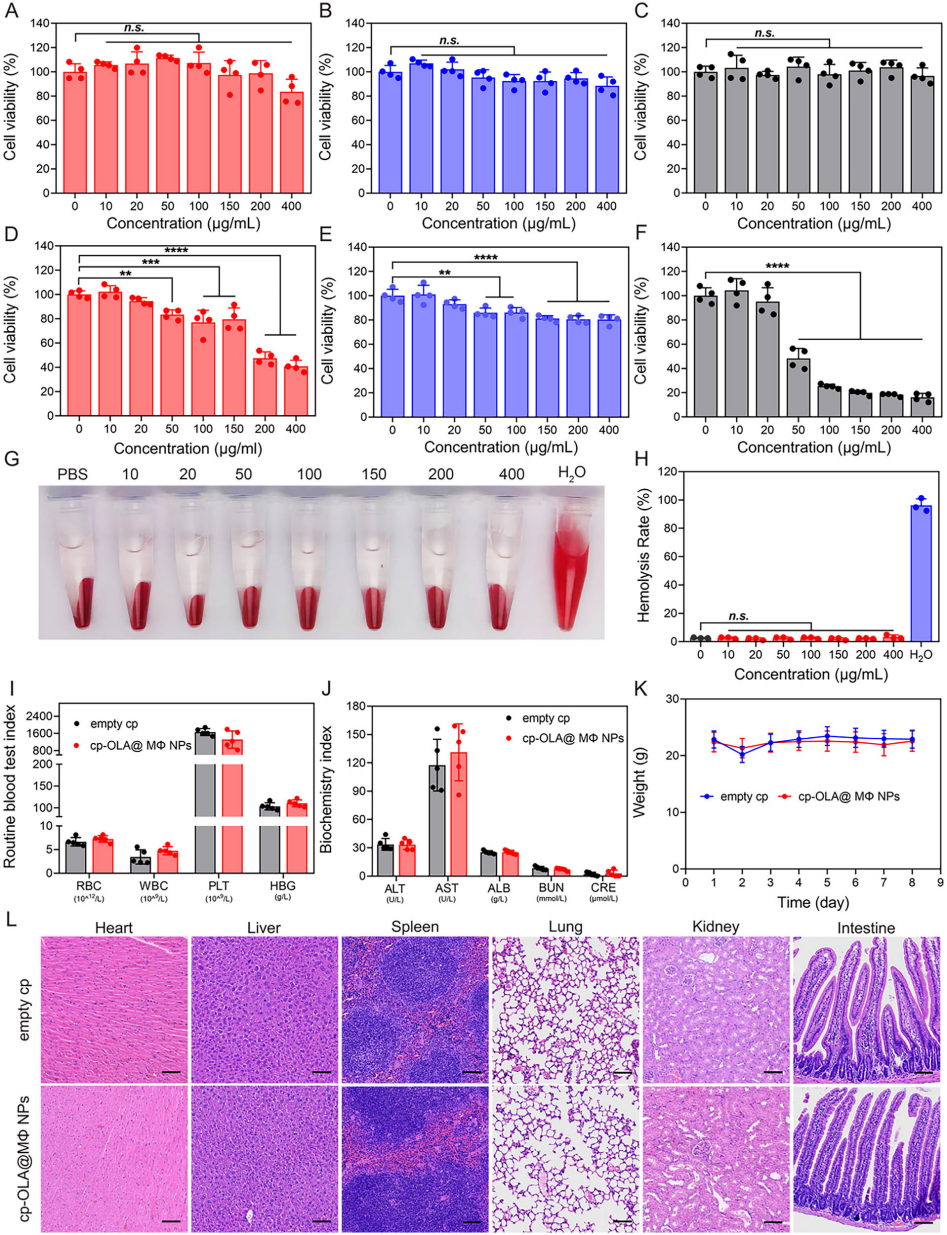
Biosafety assessment of OLA@MΦ NPs and cp‐OLA@MΦ NPs. A–C) Cell viability of HUVECs (A), L929 (B), and IEC‐6 (C) after 24 h incubation with varying concentrations of OLA@MΦ NPs (CCK‐8 assay) (*n* = 4). D–F) Cell viability analysis of HUVECs (D), L929 (E), and IEC‐6 (F) after 24 h incubation with different concentrations of OLA (CCK‐8 assay) (*n* = 4). G,H) Hemolysis analysis of fresh RBCs after 24 h incubation with different concentrations of OLA@MΦ NPs (*n* = 3). I,J) Routine blood tests and plasma biochemical parameters in healthy mice 48 h post‐oral administration of cp‐OLA@MΦ NPs (equivalent to 10 mg·kg⁻¹ OLA) or empty cp (*n* = 5). K) Body weight in healthy mice following oral administration of cp‐OLA@MΦ NPs (equivalent to 10 mg·kg⁻¹ OLA) or empty cp (*n* = 5). L) HE staining of various organs from healthy mice 48 h after oral administration of cp‐OLA@MΦ NPs (equivalent to 10 mg·kg⁻¹ OLA) or empty cp. Scale bar = 200 µm. Data are presented as mean ± SD. Statistical analysis was performed using one‐way ANOVA. **p < 0.01; ***p < 0.001; ****p < 0.0001; n.s., not significant.

The biosafety and toxicity of cp‐OLA@MΦ NPs were evaluated both in vitro and in vivo to assess their potential for therapeutic use. After 24‐h coincubation of OLA@MΦ NPs with normal cells (HUVEC, L929, and IEC‐6 cell lines), no significant reduction in cell viability was observed, even at a concentration of 400 µg·mL⁻¹ (**Figure** **6**A–C). In contrast, free OLA significantly reduced cell viability at concentrations as low as 50 µg·mL⁻¹ (Figure 6D–F). The hemolytic activity of OLA@MΦ NPs was also assessed by coincubating them with freshly collected red blood cells from healthy mice. No hemolysis was induced after 24 hours of incubation at room temperature (Figure 6G,H).

Following oral administration of cp‐OLA@MΦ NPs (equivalent to 10 mg·kg⁻¹ OLA) in healthy mice, routine blood indices, plasma biochemical markers, body weight changes, and histopathological alterations in vital organs were analyzed. No significant differences in red blood cell (RBC), white blood cell (WBC), or platelet (PLT) counts, nor hemoglobin (HBG) levels, were observed between the cp‐OLA@MΦ NP‐treated and empty cp groups at 48 h post‐administration (Figure 6I). These results indicated that cp‐OLA@MΦ NPs did not damage the hematopoietic system in healthy mice. Plasma biochemical markers, including albumin (ALB), alanine transaminase (ALT), aspartate transaminase (AST), blood urea nitrogen (BUN), and creatinine (CRE), did not show significant abnormalities (Figure 6J). Body weight also remained unchanged between the cp‐OLA@MΦ NPs‐treated and empty cp groups (Figure 6K). Histopathological examination of key organs (heart, liver, spleen, lung, kidney, and intestine) 48 h after oral administration revealed no evidence of tissue damage (Figure 6L).

## 3 Discussion

Sepsis remains a major global health challenge, characterized by high incidence and mortality rates.^[^ ^35^ ^]^ Sepsis‐induced intestinal injury plays a critical role in the progression of sepsis and is a leading cause of multi‐organ failure and poor patient outcomes.^[^ ^36^ ^]^ Current treatments are inadequate in effectively protecting intestinal barrier function,^[^ ^37^ ^]^ underscoring the urgent need for novel strategies to preserve the intestinal barrier. This study explores the potential of cp‐OLA@MΦ NPs to prevent sepsis‐induced intestinal damage and investigates the underlying mechanisms, particularly focusing on the role of parthanatos (Scheme 1).

The development of cp‐OLA@MΦ NPs represents a promising strategy that targets both the inflammatory response and parthanatos, a form of cell death triggered by DNA damage.^[^ ^38^ ^]^ These nanoparticles acquire the ability to neutralize inflammatory mediators through the integration of macrophage membranes, thereby enhancing their therapeutic efficacy. Our findings indicate that cp‐OLA@MΦ NPs effectively neutralized key pro‐inflammatory cytokines such as TNF‐α, IL‐1β, and IL‐6, all of which are closely associated with the pathophysiology of sepsis (Figure 1F–J).^[^ ^39^ ^]^

Moreover, cp‐OLA@MΦ NPs were shown to inhibit parthanatos, which is initiated by the overactivation of PARP‐1, leading to the accumulation of PAR, mitochondrial dysfunction, and subsequent cell death. By encapsulating olaparib, a potent PARP‐1 inhibitor, cp‐OLA@MΦ NPs effectively mitigated excessive PARP‐1 activation and DNA damage in intestinal epithelial cells. This resulted in reduced cell death and preservation of intestinal barrier function (Figure 2 and Figures S1 and S2, Supporting Information). Furthermore, cp‐OLA@MΦ NPs significantly decreased bacterial translocation (Figure 5L), a key contributor to systemic inflammation and septic shock.^[^ ^40^ ^]^ By maintaining the integrity of the intestinal barrier, these nanoparticles prevent bacterial migration from the intestine into the bloodstream, thereby reducing the risk of further complications in sepsis. This dual action—anti‐inflammatory and anti‐parthanatos—is essential for protecting the intestine during sepsis.

One key advantage of the cp‐OLA@MΦ NP formulation is its ability to deliver the therapeutic agent, olaparib, directly to the target site in the intestine. The pH‐responsive coating of EUDRAGIT L 30 D‐55 ensures that the nanoparticles remain intact in the acidic stomach environment, with targeted release occurring in the intestine.^[^ ^41^ ^]^ This targeted drug delivery system significantly enhances the bioavailability and efficacy of the therapeutic agent while minimizing systemic exposure and potential side effects.

## 4 Conclusion

In conclusion, the successful development of cp‐OLA@MΦ NPs demonstrates their significant potential for treating sepsis. This formulation facilitates targeted delivery to the intestine and responsive release based on gastrointestinal pH changes. Treatment with cp‐OLA@MΦ NPs resulted in a marked recovery of the structure and function of the intestine and multiple organs in septic mice. The therapeutic effects were primarily characterized by substantial anti‐inflammatory activity, organ protection, and preservation of intestinal barrier function. Overall, cp‐OLA@MΦ NPs offer a promising strategy for sepsis treatment by targeting the intestine, maintaining intestinal barrier integrity, and modulating the inflammatory response.

## 5 Experimental Section

### Reagent

Cell culture‐related reagents, including Dulbecco's Modified Eagle's Medium (DMEM), fetal bovine serum (FBS), penicillin/streptomycin, and PBS, were purchased from Gibco Life Technologies. Poly(lactic‐co‐glycolic acid) (PLGA) (50:50), olaparib, and DiR fluorescent dye were obtained from MedChemExpress. Lipopolysaccharide (LPS), *tris*‐HCl, MgCl_2_, and KCl were purchased from Sigma‐Aldrich. BCA assay, Coomassie Blue, RIPA lysis buffer, chromogenic LAL endotoxin assay kit, cell proliferation kit (CCK‐8 assay), Annexin V‐FITC apoptosis detection kit, Comet Assay Kit, and Hoechst were purchased from Beyotime Biotechnology. Tetrahydrofuran (THF), NaCl, KH_2_PO_4_, NaOH, FITC‐dextran, trypsin, pepsin, and *N,N*‐dimethylformamide were purchased from Shanghai Aladdin Biochemical Technology. Phenylmethylsulfonyl fluoride (PMSF), trypsin‐EDTA solution, and 4% paraformaldehyde were obtained from Solarbio. A capsule, capsule loading kit, and capsule dosing syringe were purchased from Shanghai Yuyan. EUDRAGIT L 30 D‐55 was obtained from Evonik. SDS–PAGE gel was obtained from GenScript. A protein loading buffer was obtained from Coolaber. Mouse TNF‐α, IL‐1β, and IL‐6 ELISA kits were purchased from Dakewe Biotech Co., Ltd. Triton X‐100 was obtained from Biosharp. Mouse anti‐mouse β‐actin, rabbit anti‐mouse IL‐1R2, rabbit anti‐mouse TNFR1, rabbit anti‐mouse TLR4, rabbit anti‐mouse AIF, rabbit anti‐mouse PARP1 and Goat Anti‐Rabbit IgG H&L (Alexa Fluor 488) were obtained from Abcam. Rabbit anti‐mouse IL‐6R, rabbit anti‐mouse ZO‐1, and rabbit anti‐mouse E‐cadherin were obtained from Proteintech. Anti‐rabbit IgG HRP‐linked antibodies and anti‐mouse IgG HRP‐linked secondary antibodies were obtained from Cell Signaling Technology. Mouse anti‐mouse PAR was obtained from Enzo Life Sciences. Rat DAO kit, rat iFABP kit, mouse DAO kit, and mouse iFABP kit were purchased from ELK Biotechnology. d‐lactate kit was obtained from Elabscience. An electron microscope fixative was obtained from ASPEN. Precision Plus Protein Dual Color Standards markers were obtained from Bio‐Rad.

### Cell Lines and Cell Culture

The rat intestinal epithelioid cell line (IEC‐6), the murine macrophage cell line (RAW 264.7), the mouse fibroblast cell line (L929), and the human umbilical vein endothelial cell line (HUVEC) were purchased from the American Type Culture Collection. IEC‐6, HUVEC and L929 cells were cultured in DMEM containing 10% FBS and 1% penicillin/streptomycin in a sterile, humidified incubator at 37 °C with 5% CO_2_. RAW 264.7 cells were cultured in DMEM containing 10% inactivated fetal bovine serum, 1% penicillin/streptomycin, 1% glutamine, and 1% sodium pyruvate in a sterile, humidified incubator at 37 °C with 5% CO_2_. The cells were passaged when they reached ≈ 70–80% confluency.

### Cell Membrane Derivation

After culturing RAW 264.7 cells in T175 culture dishes to 80–90% density, the cells were detached using trypsin, collected by centrifugation (300 × g, 5 min), and washed three times with PBS (300 × g, 5 min). The cell precipitate was subsequently resuspended in a solution containing 100 mm sucrose, 15 mm Tris‐HCl (pH = 7.5), 2 mm MgCl_2_, 10 mm KCl, and 20 mm PMSF, and ground 20–30 times using a homogenizer to break up the cells. Next, the cell suspension was sequentially subjected to the following centrifugation steps: first centrifugation at 800 × g for 5 min, collection of the supernatant followed by centrifugation at 3000 × g for 10 min, and again collection of the supernatant followed by centrifugation at 15 000 × g for 15 min. Finally, the supernatant was ultracentrifuged at 100 000 × g for 30 min using an ultracentrifuge (LE‐80K, Beckman Coulter). The pale white pellet of macrophage cell membranes was collected and stored at −80 °C for further use. Before use, the cell membranes were resuspended in PBS and lightly sonicated while the membrane protein content was determined using a standard BCA assay.

### Preparation of PLGA Cores and OLA@MΦ NPs

The PLGA core encapsulating olaparib was synthesized through nanoprecipitation. PLGA and olaparib were dissolved in tetrahydrofuran (THF) at concentrations of 10 mg·mL^−1^ and 1 mg·mL^−1^, respectively. The solution (1 mL) was then rapidly injected into 4 mL of water, followed by stirring for 4 hours in a fume hood to evaporate the THF, yielding OLA@NPs. For the preparation of OLA@MΦ NPs, macrophage membranes were extracted and mixed with PLGA cores at a 1:1 mass ratio. The mixture was extruded using a mini‐extruder (Avanti Polar Lipids), sequentially passed through polycarbonate filter membranes with pore sizes of 800 nm, 400 nm, and 200 nm, with each extrusion step repeated 20 times to ensure the macrophage membranes coated the PLGA core, forming the OLA@MΦ NPs. In fluorescence imaging assays, DiR was incorporated into the PLGA solution at a concentration of 2 µm prior to the addition of water.

### Preparation of cp‐OLA@MΦ NPs

The OLA@MΦ NPs were freeze‐dried for 24 h in a 10% sucrose solution using a lyophilizer (HORDE ELECTRIC). Following lyophilization, the resultant powder was encapsulated into gelatin capsules using a capsule‐filling machine. The capsules were then coated with an EUDRAGIT L 30 D‐55 solution and air‐dried for 10 min. This coating procedure was repeated three times to ensure uniform coverage.

### Characterization

The nanoparticle size (diameter, nm) and surface zeta potential (mV) were determined using a particle size and zeta potential analyzer (Litesizer 500, Anton Paar). The structures of the PLGA cores and OLA@MΦ NPs were observed using transmission electron microscopy (Tecnai G2 20 TWIN, FEI). DiR fluorescence release from cp‐OLA@MΦ NPs was measured using a fluorescence spectrophotometer (RF‐6000, Shimadzu). SDS–PAGE analysis was conducted using a multifunctional gel image analysis system (Tanon MINI Space 2000). Western blot bands were visualized using a chemiluminescent imaging system (ImageQuant 800, Amersham). DiR fluorescence in mouse intestines was recorded using a full‐spectrum animal live imaging system (AniView Phoenix). Flow cytometry was performed using an Attune NxT Flow Cytometer (Invitrogen). The absorbance for enzyme‐linked immunosorbent assay (ELISA) and CCK‐8 assay was measured with a microplate absorbance reader (iMark, Biorad).

### Characterization of Proteins Components of cp‐OLA@MΦ NPs

The protein profiles of PLGA cores, macrophage lysate, pure macrophage membranes, macrophage membrane‐coated PLGA nanoparticles (MΦ NPs), OLA@MΦ NPs, and cp‐OLA@MΦ NPs were analyzed via SDS–PAGE. Protein concentrations were quantified using a BCA assay kit according to the manufacturer's protocol. Samples were dissolved in protein loading buffer, heated at 100 °C for 10 min, and 20 µg of protein were loaded per well onto SurePAGE Bis‐Tris 12% 12‐well gels (GenScript). Electrophoresis was carried out in Tris‐MOPS‐SDS running buffer using the Mini‐PROTEAN Tetra electrophoresis system (Bio‐Rad). Upon completion of electrophoresis, gels were stained with Coomassie Blue solution for 1 h, and protein bands were visualized using a multi‐functional gel image analysis system (Tanon MINI Space 2000).

For Western blot analysis, MΦ NPs, OLA@MΦ NPs, and cp‐OLA@MΦ NPs, each at a protein concentration of 2.0 mg·mL^−1^, were subjected to SDS–PAGE and loaded onto SurePAGE Bis‐Tris 4–20% 12‐well gels (GenScript) for electrophoresis. Proteins were then transferred to polyvinylidene fluoride (PVDF) membranes (Merck Millipore) using a transfer buffer. The membranes were blocked with 5% milk (Biosharp) for 1 h and incubated overnight at 4 °C with primary antibodies (rabbit anti‐mouse IL‐1R, rabbit anti‐mouse IL‐6R, rabbit anti‐mouse TNF‐αR, and rabbit anti‐mouse TLR4). Following primary antibody incubation, the membranes were incubated at room temperature for 1 h with an anti‐rabbit IgG HRP‐linked secondary antibody. Protein bands were visualized using a chemiluminescence imaging system (ImageQuant 800, Amersham).

### Quantification of In Vitro Cytokine and LPS Binding

To assess cytokine and LPS binding, IL‐1β (500 pg·mL^−1^), IL‐6 (500 pg·mL^−1^), TNF‐α (500 pg·mL^−1^), and LPS (250 ng·mL^−1^) were incubated with different concentrations of OLA@MΦ NPs (125, 250, 500, 1000, and 2000 µg·mL^−1^), respectively, at 37 °C for 2 h. After incubation, the supernatant was collected by centrifugation at 16000 × g for 10 min. The concentrations of unbound cytokines (IL‐1β, IL‐6, and TNF‐α) in the supernatants were measured by ELISA, while unbound LPS levels were determined using a Chromogenic LAL Endotoxin Assay Kit. Bound cytokine and LPS levels were calculated by subtracting the concentrations of the unbound forms from their initial concentrations. Data analysis was performed using GraphPad Prism 8 software for nonlinear curve fitting.

### In Vitro and In Vivo Release Evaluation of OLA@MΦ NPs

To assess the protective efficacy of the capsule against drug degradation in the gastric environment, DiR‐labeled OLA@MΦ NPs were lyophilized and encapsulated in the capsule coated with EUDRAGIT L 30 D‐55. The capsules were incubated in either simulated gastric fluid (SGF, pH = 2) or simulated intestinal fluid (SIF, pH = 6.8) under stirring conditions at 800 rpm at room temperature. Samples were collected at 0, 1, 2, 5, 10, 15, 30, 60, and 120 min. Fluorescence intensity, indicative of DiR signal (excitation/emission = 748/780 nm), was measured using a fluorescence spectrophotometer (RF‐6000, Shimadzu). To evaluate capsule integrity in the stomach and targeted drug release in the intestine, DiR‐labeled cp‐OLA@MΦ NPs were administered orally to 7‐week‐old male C57BL/6 mice using a capsule delivery syringe. Mice were sacrificed at 0, 2, 4, 6, and 24 h post‐administration, and the entire gastrointestinal (GI) tract was harvested for analysis. Drug biodistribution was imaged and evaluated using the AniView Phoenix system.

### In vitro Experiments

To validate the protective effect of IEC‐6 cells against H_2_O_2_‐induced damage, cells were divided into five groups: control, PBS, OLA, MΦ NPs, and OLA@MΦ NPs. IEC‐6 cells were first incubated with PBS, OLA, MΦ NPs, or OLA@MΦ NPs (equivalent to 5 µg·mL^−1^ of OLA) for 30 min, followed by exposure to 300 µm H_2_O_2_. Subsequent evaluations included cell viability, expression of PARP1, PAR, AIF, ZO‐1, E‐cadherin, and the levels of DAO and iFABP in the cell supernatant.

### Cell Viability Assay

IEC‐6 cells were seeded in 96‐well plates at 5 × 10^3^ cells/well. After cell adhesion, treatments were applied, and after 1 h, the culture medium was replaced with a CCK‐8 working solution and incubated for 1.5 h. The optical density (OD) was measured at 450 nm using a microplate absorbance reader (BIO‐RAD). Results were expressed as the average percentage of viable cells in the treated group relative to the untreated control group.

IEC‐6 cells were seeded in 6‐well plates at a density of 5 × 10⁴ cells/well. Upon adhesion, the cells underwent the corresponding treatments. After 24 h, cells were harvested, washed with PBS, and stained using the Annexin V‐FITC apoptosis detection kit. Apoptosis was assessed by flow cytometry (Attune NxT Flow Cytometer, Invitrogen), and data analysis was performed using FlowJo software.

### DNA damage assessment

DNA damage was assessed using the comet assay kit following the manufacturer's protocol. Cells were harvested and resuspended in PBS to a concentration of 1 × 10⁶ cells/ml. A layer of normal melting point agarose was spread onto a slide, and after solidification, the cell suspension was mixed with low melting point agarose and added dropwise to the slide. The slide was cooled at 4 °C for 10 min. Next, the slide was incubated in lysis buffer for 2 h, followed by PBS washing for 3 min. The slide was then placed in an alkaline electrophoresis buffer for 1 h to allow DNA unwinding, followed by electrophoresis at 25 V for 30 min. After electrophoresis, the slide was immersed in neutralizing buffer for 5 min, stained for 10 min, and visualized using an inverted fluorescence microscope (Olympus, IX73). Data analysis was performed using the Comet Assay Software Project (CASP, SourceForge).

### Immunofluorescence Stain

Immunofluorescence staining was performed to detect ZO‐1, E‐cadherin, and AIF proteins in IEC‐6 cells. After 4 h of treatment, cells were washed with PBS, fixed with 4% paraformaldehyde for 15 min at room temperature, and treated with 0.1% Triton X‐100 for 15 min, followed by blocking with 5% BSA for 1 h. Cells were then incubated overnight at 4 °C with primary antibodies (rabbit anti‐mouse ZO‐1, rabbit anti‐mouse E‐cadherin, and rabbit anti‐mouse AIF). Afterward, cells were incubated with goat anti‐rabbit IgG (H+L) Alexa Fluor 488 secondary antibody for 1 h at room temperature. Nuclei were stained with Hoechst for 10 min. Immunofluorescence signals were observed using a confocal microscope (Suny CSIM110).

### Western Blot

Western blot analysis was conducted to detect ZO‐1, E‐cadherin, PARP1, and PAR proteins. Cells and tissues were lysed on ice for 10 min in RIPA buffer containing PMSF. The lysate was centrifuged at 14 000 rpm for 10 min at 4 °C to collect the supernatant. Protein concentration was determined using a BCA protein assay kit, and the samples were mixed with protein loading buffer and heated at 100 °C for 10 min. An aliquot (20 µg) of each protein sample was separated by SDS–PAGE, followed by transfer to a PVDF membrane. The membrane was blocked with 5% skim milk for 1 h and incubated overnight at 4 °C with primary antibodies: rabbit anti‐mouse ZO‐1, rabbit anti‐mouse E‐cadherin, rabbit anti‐mouse PARP1, and mouse anti‐mouse PAR. After washing, the membrane was incubated for 1 h at room temperature with HRP‐linked secondary antibodies: anti‐rabbit IgG for ZO‐1, E‐cadherin, and PARP1, and anti‐mouse IgG for PAR. Protein bands were visualized using a chemiluminescence imaging system (ImageQuant 800, Amersham).

### Enzyme‐linked Immunosorbent Assay (ELISA)

DAO and iFABP levels in IEC‐6 cell culture supernatants were quantified using rat‐specific kits, following the manufacturer's guidelines. Serum concentrations of IL‐1β, IL‐6, TNF‐α, DAO, iFABP, and D‐lactate were determined using corresponding mouse‐specific assay kits, also according to the manufacturer's protocol.

To detect IL‐1β, IL‐6, TNF‐α, DAO, and iFABP, samples, and standards were added to the plate wells, followed by the biotinylated antibody. The plate was incubated at 37 °C for antigen‐antibody binding, washed with buffer, and then incubated with Streptavidin‐HRP at 37 °C. After another wash, 3,3′,5,5′‐Tetramethylbenzidine (TMB) substrate solution was added for color development. Following the color reaction, a stop solution was introduced, and absorbance was measured at 450 nm using a microplate reader (iMark, Bio‐Rad). For D‐lactate, samples and standards were added to the wells, followed by sequential addition of enzyme stock solution and chromogenic agent. After a 37 °C incubation, a stop solution was added, and absorbance was measured at 530 nm with the same microplate reader.

### In Vitro Permeability Study

IEC‐6 cells were seeded in 24‐well transwell plates (Corning) with a 0.4 µm pore size. Upon reaching 80% confluence, the cells were pretreated for 30 min with different conditions. Subsequently, the cells were exposed to 300 µm H_2_O_2_ for 24 h. After treatment, the medium was aspirated, and both the upper and lower chambers were washed with PBS. FITC‐dextran (1 mg·mL^−1^) dissolved in a complete culture medium (300 µL) was added to the upper chamber. After 24 h of incubation at 37 °C, the liquid in the lower chamber was collected, and the FITC signal was measured using a fluorescence spectrophotometer with excitation/emission wavelengths of 480/520 nm.

### Establishment of a Septic Mouse Model

Animal experiments were approved by the Experimental Animal Ethics Committee of Renmin Hospital of Wuhan University (Approval No. 20210303A), in compliance with relevant ethical guidelines. Male C57BL/6 mice (6‐8 weeks old) were sourced from Guangdong Medical Laboratory Animal Center (Guangdong, China) and housed in controlled conditions with free access to food and water under a 12 h light/12 h dark cycle. All mice were maintained in a specific pathogen‐free (SPF) environment.

Mice were randomly assigned to five groups: control group (PBS intraperitoneal injection), empty cp group, OLA group, OLA@MΦ NPs group, and cp‐OLA@MΦ NPs group. All groups, except the control group, received a 15 mg·kg⁻¹ intraperitoneal LPS injection, and 30 min prior to LPS injection, mice in each experimental group were orally administered empty capsules, OLA, OLA@MΦ NPs, or cp‐OLA@MΦ NPs (10 mg·kg⁻¹ OLA equivalent). Mice were euthanized 24 h post‐induction for tissue collection. Body temperature was monitored at 0, 6, 12, and 24 hours post‐induction using a thermal imaging device (Fotric), and survival rates were recorded at 0, 12, 24, 36, and 48 h post‐induction.

### Clinical Score

At 24 h post‐LPS stimulation, clinical assessment was performed using the murine sepsis score (MSS) system (**Table** **1**).

**Table 1:** Murine sepsis score (MSS) system for evaluating clinical symptoms of mice.

Variable	Score and description
Appearance	0. Coat is smooth 1. Patches of hair piloerected 2. Majority of back is piloerected 3. Piloerection may or may not be present, the mouse appears “puffy” 4. Piloerection may or may not be present, mouse appears emaciated
Level of consciousness	0. Mouse is active 1. Mouse is active but avoids standing upright 2. Mouse activity is noticeably slowed. The mouse is still ambulant 3. Activity is impaired. Mouse only moves when provoked, movements have a tremor 4. Activity severely impaired. Mouse remains stationary when provoked, with possible tremor
Activity	0. Normal amount of activity. Mouse is involved in any of the following activities: Eating, drinking, climbing, running, fighting 1. Slightly suppressed activity. Mouse is moving around bottom of cage 2. Suppressed activity. Mouse is stationary with occasional investigative movements 3. No activity. Mouse is stationary 4. No activity. Mouse experiencing tremors, particularly in the hind legs
Response to stimulus	0. Mouse responds immediately to auditory stimulus or touch 1. Slow or no response to auditory stimulus, strong response to touch (moves to escape) 2. No response to auditory stimulus, moderate response to touch (moves a few steps) 3. No response to auditory stimulus, mild response to touch (no locomotion) 4. No response to auditory stimulus. Little or no response to touch. Cannot right itself if pushed over
Eyes	0. Open 1. Eyes not fully open, possibly with secretions 2. Eyes at least half closed, possibly with secretions 3. Eyes half closed or more, possibly with secretions 4. Eyes closed or milky
Respiration rate	0. Normal, rapid mouse respiration 1. Slightly decreased respiration (rate not quantifiable by eye) 2. Moderately reduced respiration (rate at the upper range of quantifying by eye) 3. Severely reduced respiration (rate easily countable by eye, 0.5 s between breaths) 4. Extremely reduced respiration (>1 s between breaths)
Respiration quality	0. Normal 1. Brief periods of labored breathing 2. Labored breathing, no gasping 3. Labored with intermittent gasping 4. Gasping

### Histological Study

For H&E analysis, the heart, liver, lungs, kidneys, and intestinal tissues were collected from mice and fixed in 4% paraformaldehyde for 24 h. The tissues were then embedded in paraffin and sectioned into 5 µm thick slices. These sections were stained with H&E (Beyotime Biotechnology) and observed under a microscope.

For immunofluorescence staining, fresh intestinal tissue was rapidly frozen in liquid nitrogen for 5 min, then stored at –80 °C. The tissue was embedded and sectioned into 5 µm thick slices, which were rewarmed at room temperature for 30 min and fixed with 4% paraformaldehyde for 30 min. Antigen retrieval was performed, followed by blocking with a 10% BSA solution for 30 min at room temperature. The primary antibody, rabbit anti‐mouse ZO‐1, was added and incubated overnight at 4 °C. Afterward, goat anti‐rabbit IgG (Cy3) secondary antibody was added and incubated at room temperature for 1 h. TSA reagent was then applied and incubated for 20 min at room temperature. The sections were blocked again with 10% BSA solution for 10 min before applying the primary antibody, rabbit anti‐mouse E‐cadherin, and incubating overnight at 4 °C. The secondary antibody, goat anti‐rabbit IgG (Alexa Fluor 488), was applied and incubated at room temperature for 1 h, followed by a second incubation with TSA reagent for 20 min. Nuclei were stained with Hoechst for 10 min, and the sections were examined using a fluorescence microscope. For transmission electron microscopy (TEM), intestinal tissue was cut into 1 mm^3^ pieces and fixed in an electron microscope fixative for 4 h. After dehydration, permeabilization, and embedding, ultra‐thin sections (60–80 nm) were prepared using an ultramicrotome. The sections were then double‐stained with uranyl acetate and lead citrate, and images were obtained using a transmission electron microscope (Tecnai G2 20 TWIN, FEI).

### Intestinal Permeability Assay

60 min prior to euthanizing the mice, 200 µL of 10 mg·mL⁻¹ Evan's blue solution (Macklin) was injected via the tail vein. The intestinal tissue was then collected and photographed. Additionally, 5 mL of PBS was used for peritoneal lavage, and the collected lavage fluid was analyzed for colony‐forming units (CFU).

### Biosafety Study

Different concentrations of OLA and OLA@MΦ NPs (0, 10, 20, 50, 100, 150, 200, 400 µg·mL⁻¹, based on OLA concentration) were applied to three types of normal cells (HUVEC, L929, and IEC‐6). After 24 h of co‐incubation, cell proliferation, and viability were assessed using a CCK‐8 assay kit.

For the hemolysis experiment, different concentrations of OLA@MΦ NPs (10, 20, 50, 100, 150, 200, 400 µg·mL⁻¹ based on OLA concentration, with PBS and H_2_O as controls) were co‐incubated with fresh mouse red blood cells (1 × 10^8^ mL⁻¹) for 24 h, followed by centrifugation at 800 × g for 5 min. The appearance of the supernatant was recorded, and absorbance at 595 nm was measured to assess the extent of hemolysis.

For the in vivo biosafety study, six‐week‐old male C57BL/6 mice were randomly divided into two groups and allowed to acclimate for one week before the start of the experiment. Empty capsules or cp‐OLA@MΦ NPs (OLA concentration 10 mg·kg⁻¹) were administered orally using a capsule dosage syringe. After 48 h, blood was collected for analysis of hematological parameters, including red blood cells (RBC), white blood cells (WBC), platelets (PLT), hemoglobin (HGB), and biochemical markers such as alanine aminotransferase (ALT), aspartate aminotransferase (AST), albumin (ALB), blood urea nitrogen (BUN), and creatinine (CRE). Major organs (heart, liver, spleen, lung, kidney, and intestine) were collected for histological analysis. One capsule was given to each group of mice daily for 8 consecutive days throughout the treatment period, and the mice were monitored for body weight changes.

### Statistical Analysis

Quantitative data were presented as mean ± standard deviation (SD) from three or more independent experiments. For multiple group comparisons, one‐way analysis of variance (ANOVA) was used. Statistical analyses were performed using GraphPad Software version 8. Statistical significance was indicated by asterisks: *p < 0.05; **p < 0.01; ***p < 0.001; n.s., not significant.

## Conflict of Interest

The authors declare no conflict of interest.

## Author Contributions

Y.Y., B.L., Q.G., M.W., and H.M. contributed equally to this work. Y.Y., B.L., and Q.G. designed the research. Y.Y., B.L., Q.G., M.W., H.M., J.B., C.M., X.X., Y.G., L.X., X.L., Wei W., Y.W., J.W., H.W., Y.F., Y.Z., P.L., H.S., F.M., Y.J., H.D., X.F. and Wenying W. performed the research. L.Z., X.D., H.Z., and Y.L. provided professional support for animal studies. All authors analyzed and interpreted the data. Y.Y., B.L., and Q.G. wrote the paper.

## Supporting information



Supporting Information

## Data Availability

The data that support the findings of this study are available from the corresponding author upon reasonable request.;
