# Supplementary material for: Intestine‐Decipher Engineered Capsules Protect Against Sepsis‐induced Intestinal Injury via Broad‐spectrum Anti‐inflammation and Parthanatos Inhibition
Source: Adv Sci (Weinh). 2025 Jan 21;12(10):2412799. doi: 10.1002/advs.202412799 (PMC11904959; doi:10.1002/advs.202412799)
Supplement: Supplementary file 1 — Supporting Information [file ADVS-12-2412799-s001.docx]

Intestine-decipher engineered capsules protect against sepsis-induced intestinal injury via broad-spectrum anti-inflammation and Parthanatos inhibition

Yan Yan^1‡^, Bin Li^2‡^, Qiuxia Gao^3‡^, Miao Wu^1‡^, Hua Ma^3‡^, Jiawei Bai^1^, Chengtai Ma^1^, Xinyu Xie^1^, Yuan Gong^4^, Lingqi Xu^4^, Xiaoxue Li^5^, Wei Wang^5^, Yanqiu Wu^5^, Jiamei Wang^3^, Huanhuan Wang^3^, Yi Feng^5^, Yunlong Zhang^1^, Peiran Li^6^, Huimin Shi^5^, Fei Ma^2^, Yue Jia^2^, Han Duan^6^, Xinting Fu^6^, Wenying Wang^3^, Liying Zhan^1^*, Xianjin Du^1^*, Huiting Zhou^4^* and Yuhui Liao^2, 3^*

1. Department of Critical Care Medicine, Renmin Hospital of Wuhan University, Wuhan 430060, China.

2. School of Inspection, Ningxia Medical University, Yinchuan 750004, Ningxia, China.

3. Institute for Engineering Medicine, Kunming Medical University, Kunming 650500, China.

4. Institute of Pediatric Research, Children's Hospital of Soochow University, Suzhou, 215025, China.

5. Molecular Diagnosis and Treatment Center for Infectious Diseases, Dermatology Hospital of Southern Medical University, Guangzhou 510091, Guangdong, China.

6. Department of Microbiology, School of Public Health, Southern Medical University, Guangzhou 510515, China.

*Corresponding Authors: liaoyh8@mail.sysu.edu.cn (Y. Liao); htzhou@suda.edu.cn (H. Zhou); duxianjin@whu.edu.cn (X. Du); zhanliying@whu.edu.cn (L. Zhan)

‡These authors contributed equally to this work.


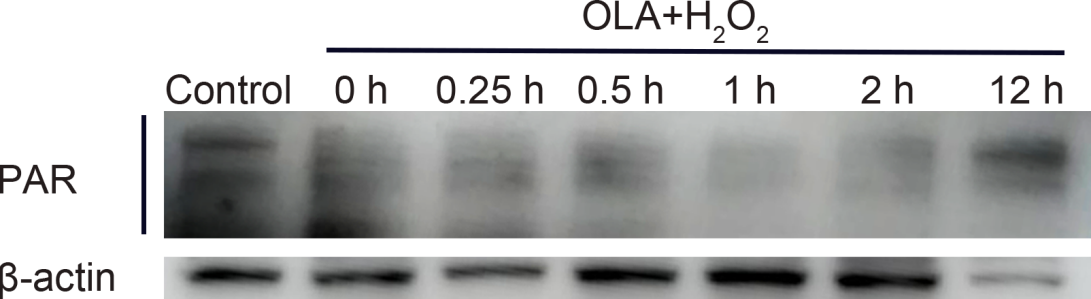


**Figure S1.** Western blot analysis of PAR expression at different times after exposure to 300 μM H_2_O_2_ after OLA pretreatment.


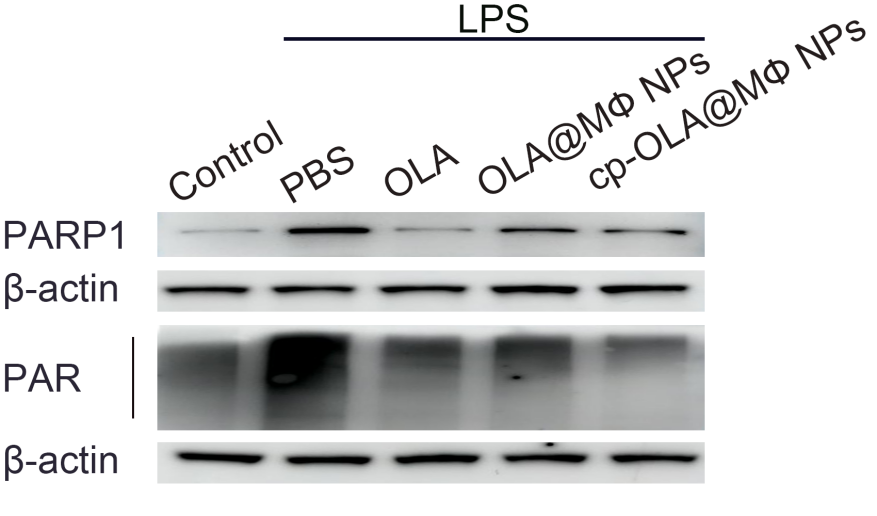


**Figure S2.** Western blot analysis of intestinal PARP1 and PAR expression of septic mice following various treatments


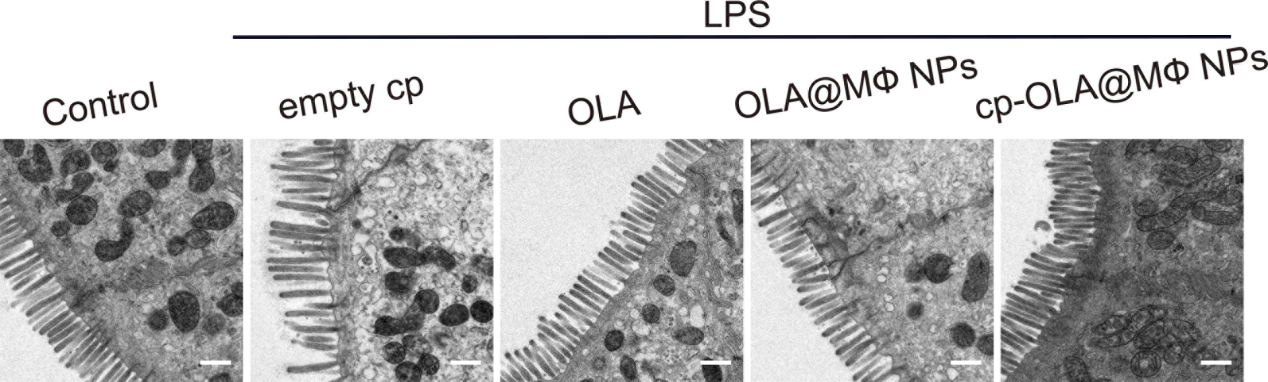


**Figure S3.** TEM images of the colon. Scale bar = 1.5 μm.
